# Lithium Chloride rescues Monensin-potentiated Wnt signaling inhibition in inflammatory bone loss in rats

**DOI:** 10.1590/0103-644020256475

**Published:** 2025-11-21

**Authors:** Anderson Chagas, Sthefane Gomes, Denis Oliveira, Khalil Viana, Jennifer Chaves, Conceição Martins, Vanessa Costa, Gisele Angelino, Sislana Azevedo, Diego Almeida, João Martins Sena, Delane Gondim, Renata Leitão, Mirna Marques, Paula Goes

**Affiliations:** 1 Post-Graduate Program in Morphofunctional Sciences, Department of Morphology, Federal University of Ceará, Fortaleza, Ceará, Brazil.; 2 Post-Graduate Program in Dentistry, Department of Clinical Dentistry, Federal University of Ceará, Fortaleza, Ceará, Brazil; 3 Department of Clinical Dentistry, Federal University of Ceará, Fortaleza, Ceará, Brazil; 4 Department of Morphology, Federal University of Ceará, Fortaleza, Ceará, Brazil; 5 Fundação Oswaldo Cruz- Ceará, Eusébio, Ceará, Brazil; 6 School of Medicine, Federal University of Ceará, Sobral, Ceará, Brazil.; 7 Department of Pathology and Legal Medicine, Federal University of Ceará, Fortaleza, Ceará, Brazil

**Keywords:** Lithium, Monensin, WNT pathway, Periodontitis, Bone Resorption

## Abstract

Wnt signaling has been related to inflammatory bone loss. The study aimed to evaluate the effect of Lithium Chloride (LiCl) on animals submitted to Monensin-potentiated periodontal bone loss. For that, Wistar rats were submitted to experimental periodontitis (EP) and received either 0.1ml/200g corn oil (vehicle) daily; or Monensin (Mon), receiving 10 mg/kg of Mon daily; or LiCl, receiving 150mg/kg of LiCl on alternate days; or Mon+LiCl. The Naïve (N) group was not submitted to any treatment. After euthanasia, maxillae were collected for macroscopic and histological analyses, and bone markers and Wnt signaling were expressed. Molecular docking assays were used to assess the affinity between Mon and WNT pathway components. It was seen that EP caused bone loss and inflammation in the periodontium, which was potentiated by Mon. LiCl protected bone and rescued the deleterious effects of Mon marked by a reduction in bone loss, an increase in osteoblasts number (39%), and Runx2 (70%) and OPG (68%) gene expressions, concomitant with a reduction of osteoclasts in number (32%) and function (27%). LiCL increased beta-catenin's gene and protein expressions in animals with EP receiving Mon. In summary, LiCl rescued the deleterious effect of Mon on bone in animals with EP and stands as a pharmacological tool to counteract the potent inhibition of the Wnt pathway.

**Figure:**
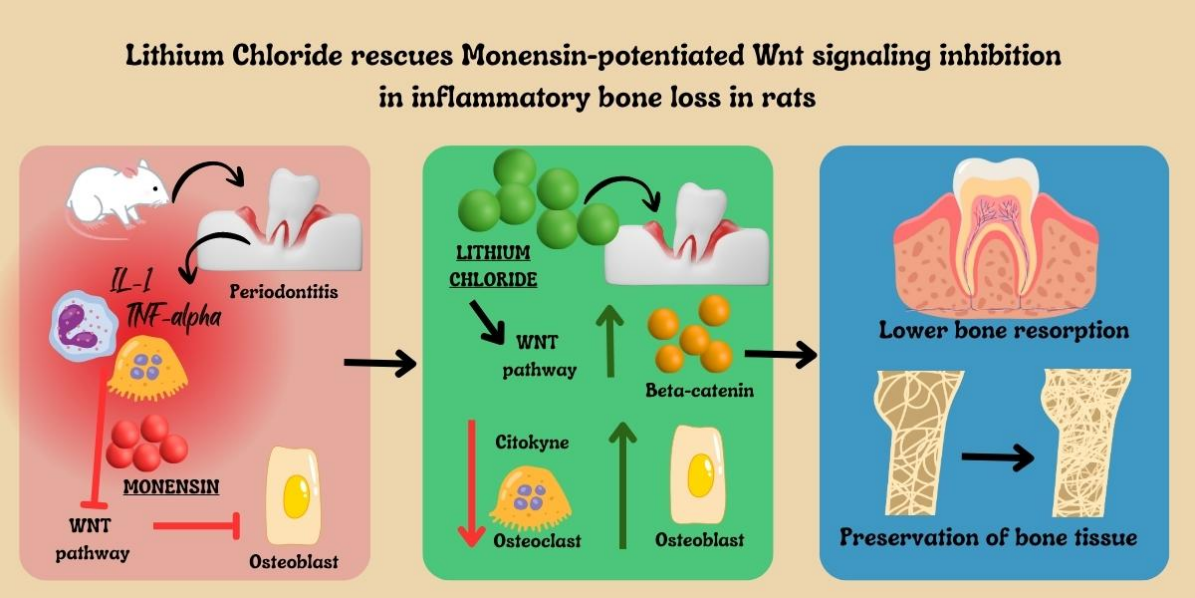

## Introduction

Periodontitis is a highly prevalent chronic inflammatory disease. Its etiology is multifactorial and complex. Periodontal pathogenic bacteria stimulate inflammation, leading to bone loss ^1^. This inflammatory bone loss can be explained by inflammatory infiltrate and cytokines unbalancing molecular pathways, such as Wnt signaling ^2^.

WNT/beta-catenin pathway is a well-known pathway related to cell proliferation and cancer that has also been highlighted as a regulator of bone metabolism, involved with the differentiation of osteoblasts ^3^. The interaction between WNT proteins and their receptors (FZD and LRP5/6) inhibits the action of Glycogen Synthase Kinase 3 Beta (GSK3b), favoring the accumulation of β-catenin in the cytoplasm which can then translocate to the nucleus, stimulating the expression of genes such as Runx2 and osteoprotegerin (OPG) related to osteoblastogenesis ^4^. Physiologically, this pathway is inhibited by Dickkopf-1 (DKK-1) and Sclerostin (SOST) ^5^. Our group has reported that the inflammatory process increases DKK-1 expression, reducing bone formation in periodontitis models and the jaws' osteonecrosis ^6^ ^,^ ^7^ ^,^ ^8^.

Knowing, therefore, the importance of the WNT pathway for bone metabolism, it becomes interesting to evaluate the effect of drugs that modulate this pathway. LiCl has been used to prevent mood swings and suicide and is also used as an adjunct in the treatment of depression ^9^. However, it has also been shown to be associated with the formation of bone tissue ^10^ precisely due to the inhibition of GSK3b, favoring the accumulation of β-catenin in the nucleus and, therefore, stimulating osteoblastogenesis ^11^. Monensin (Mon), on the other hand, is a natural compound isolated from *Streptomyces cinnamonensis* and belongs to the group of ionophore antibiotics that bind to cations such as Na+, K+, and Li+. Mon exhibits a broad spectrum of biological activities such as antimicrobial, antiproliferative, antiparasitic, and antiviral ^12^. However, more recently, Monensin has been studied as a drug with anti-cancer activity ^12^, showing positive effects both *in vitro* and *in vivo* assays against melanoma cells ^13^, acute myeloid leukemia ^14^, and prostate cancer ^15^. Monensin blocks the phosphorylation of LRP6, inducing its degradation, and more especially, it inhibits β-catenin activity, thus turning off the WNT pathway ^16^.

Therefore, knowing that the blockage of Wnt signaling contributes to inflammatory bone loss and that bone resorption can be potentiated by the use of Wnt inhibitors, such as Monensin, we have decided to investigate if LiCl can rescue Wnt pathway activation since it was previously reported that other types of GSK3b inhibitors have failed ^16^.

## Materials and methods

### Study design and ethical aspects

This is an experimental study using animal models submitted to periodontitis. The experiments had their protocols carried out based on the recommendations of ARRIVE guidelines (Animal Research: Reporting In Vivo Experiments guidelines) and began shortly after approval by the Institutional Ethics Committee for Animal Use (CEUA #7128020620), which is governed by the Universal Declaration of Animal Rights (UNESCO - January 27^th^, 1978) and the International Ethical Guidelines for Biomedical Research Involving Animals (Council for International Organizations of Medical Sciences - CIOMS).

The sample size was determined considering α = 0.05 and a power of 0.8. Thus, 6 animals per group were required. Alveolar bone loss > 3.5 mm was considered as the primary outcome variable.

### Ligature-induced periodontitis model and Experimental groups

This was a preclinical randomized and blinded study. Ninety male Wistar rats (*Rattus novergicus*), with a body mass of approximately 200 g and 12 weeks old, were used for the study. All animals received balanced commercial food and water *ad libitum* and remained under the same environmental conditions of light/dark cycles of 12 hours and room temperature of 22°C throughout the experiment. There was no animal exclusion.

Periodontitis was induced by placing a 3.0 nylon suture around the 2^nd^ upper left molar ^17^ in a rat, previously anesthetized with Ketamine (80 mg/kg) and Xylazine (10 mg/kg) intraperitoneally. After the placement of the ligature, a surgical knot was tied facing the buccal surface of the animal’s oral cavity. At the end of the experiment (11^th^ day), the animals were euthanized by an overdose of Ketamine (240 mg/kg) and Xylazine (30 mg/kg), administered IP.

After two weeks of acclimation to the laboratory environment, the animals were divided in a blind and randomized manner. Randomization was performed using computer software, considering the weight of the animals. The animals were initially divided into 5 groups (n=6 animals per group):

- Naïve Group (**N**): Animals were not submitted to any treatment or procedure;
- Experimental periodontitis (**EP**) group: Animals received corn oil (vehicle) by gavage 30 min before periodontitis induction and daily for 11 consecutive days until euthanasia;
- Monensin (**Mon**) group: Animals received 10mg/kg of Monensin (Sigma-Aldrich-San Luis, Missouri, USA- No. M5273) by gavage, 30 min before the induction of periodontitis, and daily for 11 consecutive days until euthanasia ^16^;
- Lithium chloride (**LiCl**) group: animals received 150mg/kg of LiCl every other day by gavage for 11 days until euthanasia (Cequímica-Fortaleza, Ceará, Brazil) ^18^;
- Monensin+Lithium Chloride Group (**Mon+LiCl**): The animals received 10mg/kg of Mon and 150 mg/kg of LiCl every other day by gavage for 11 days until euthanasia.

Three sets of experiments (n=30 animals/set) were performed for this study. The first set was used for macroscopic analysis, the second for histopathological histomorphometric studies and immunohistochemistry assays, and the third for determining gene expression by RT-PCR. In all sets of experiments, blood samples were collected for further dosage of serum levels of DKK1 and CTx.

### Macroscopic analysis of the alveolar bone

After euthanasia, the maxillae were removed and fixed in 10% buffered formalin for 48 hours. They were then dissected and separated into hemiarches, clarified in 2.5% sodium hypochlorite for 1 minute, and stained in 1% methylene blue for 10 seconds to differentiate the bone tissue from teeth (Adapted from GOES et al. ^19^).

Subsequently, the hemimaxillae were photographed. Bone resorption was measured considering the difference between the area of cementum-enamel junction until bone crest in the region between the first and third molars from the left and right sides, using the Image J® software (NIH, Bethesda, Maryland, USA) ^17^.

### Histopathological analysis of the periodontium

A new set of experiments was performed for this analysis because the samples used for macroscopic analyses had their gingival tissue removed, and the maxillae were stained. After the euthanasia, the maxillae were fixed in 10% neutral formaldehyde for 48 hours. Then, they were decalcified in 10% EDTA, neutral pH ^19^, for four weeks. Subsequently, the material was embedded in paraffin, and 4 µm thick sections were collected and stained with hematoxylin-Eosin (HE). 

For the microscopic analysis, the region between the 1^st^ and 2^nd^ molars was considered, and scores ranging from 0 to 3 were assigned according to the intensity of the findings, considering the following aspects: presence/intensity of cellular infiltrate and state of preservation of the alveolar process and cementum ^20^. So, zero was classified as absent, and three was classified as high intensity of alteration.

### Histometric analysis of alveolar bone

For this analysis, we used the same slides as those used for the histological study. The slides presented in the same histological section were selected for dental root, interdental papilla, and interproximal bone. Images of 4 fields were obtained from the bone tissue of the interproximal region between the first and second upper left molar at 400x magnification ^21^. The images were launched in the Image J® software (NIH, Bethesda, MD, USA), and an observer blinded to the groups performed the osteoblast and osteoclast count by bone perimeter (N.Ob/B.Pm and N.Oc/B.Pm, respectively) using the Image J® software ^8^.

### Immunohistochemistry for beta-catenin

Immunohistochemistry for beta-catenin was performed using the streptavidin-biotin-peroxidase method in formalin-fixed, paraffin-embedded tissue sections (4μm thick) and mounted on poly-L-lysine coated microscope slides. The sections were deparaffinized and rehydrated through xylene and graded alcohols. After antigen retrieval, endogenous peroxidase was blocked (30 min) with 3% (v/v) hydrogen peroxide and washed in phosphate-buffered saline (PBS). Sections were incubated overnight (4°C) with anti-beta-catenin (1:200 ABCAM®, Cambridge, MA, USA). The slides were then incubated with the secondary antibody diluted 1:200 in PBS-BSA. After washing, the slides were incubated with avidin-biotin-horseradish peroxidase conjugate for 30 min, following the manufacturer's instructions. Beta-catenin was visualized with the chromogen 3,3 diaminobenzidine (DAB) after 2 min of incubation. Negative control sections were processed simultaneously as described above, but the first antibody was replaced by 5% PBS-BSA. Slides were counterstained with hematoxylin, dehydrated in a graded alcohol series, cleared in xylene, and coverslipped. The immunostained osteoblasts for beta-catenin of five different areas of each section (from four specimens per group) were quantified at 400x magnification.

### RNA isolation and quantitative PCR

In the third set of experiments, after euthanasia, the left hemimaxillae were collected, the gingival tissue was removed, and the bone tissue was macerated in liquid nitrogen using Trizol (Thermo Fischer-Waltham, Massachusetts, USA). The extracted mRNA was quantified using Nanodrop (Thermo Fischer-Waltham, Massachusetts, USA) and then transcribed using Superscript II (Invitrogen). Subsequently, the RT-PCR assay was carried out using SYBR_green as a reference (ABI 7500 Fast; Applied Biosystems). The PCR condition was 50°C for 2 minutes and 90°C for 10 minutes, then 40 cycles at 95°C for 15 seconds and 60°C for 1 minute, where the RT-PCR system at 7900HT from Applied Biosystems. The threshold cycle method ^10^ was used to calculate the results obtained, presenting them as an x-fold increase related to beta-actin. Primer sequences were as follows (Box 1)

**Box 1:**
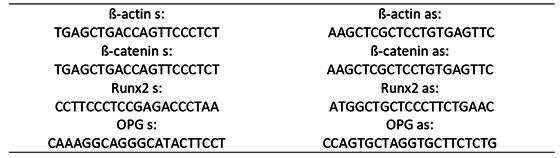
Primer sequences

### Blood collection and biochemical analysis

After anesthesia, 2ml of blood was collected from each animal by cardiac puncture at the time of euthanasia in all experimental sets. The blood was then used for biochemical analysis of Dickkopf protein 1 (DKK1) and C-telopeptide of collagen (CTx) using ELISA according to the manufacturer's guidelines Abebio® (Wuhan, China).

### Molecular docking with Wnt pathway components

The molecular docking technique was employed to analyze the interaction mode between Monensin and the proteins involved in the WNT pathway. This computational method can efficiently predict the binding mode and affinity between two molecules ^23^ ^,^ ^24^.

### Monensin and protein structure preparation

The chemical structure of Monensin, identified by ID 441145, was obtained from the PubChem database. Subsequently, the protonation state was determined using the MarvinSketch program^© (^ ^25^. Based on literature references ^16^, the β-catenin and LRP6 proteins from the WNT pathway were chosen as targets for molecular docking calculations. Their three-dimensional structures were obtained from the Protein Data Bank (PDB) with codes 1qz7 (resolution of 2.2 Å) and 3s8v (resolution of 3.1 Å), respectively.

As a pre-processing step for molecular docking, the protonation state of the proteins at pH 7.4 was determined using the PDB2PQR software ^26^. The original PDB files also removed hydrogen atoms, water molecules, and small existing molecules. Furthermore, only the E3 and E4 domains of the extracellular portion of the LRP6 protein were taken into consideration.

### Molecular docking calculations

Docking molecular assays were performed using the DockThor software ^27^ ^,^ ^28^. The structural regions were constrained to known interaction sites of other inhibitors reported in the literature, with the grid centered on these regions, the coordinates, and other parameters presented in Box 2. A total of 24 poses were generated and ranked by the binding affinity score provided by DockThor, which utilizes the DockTScore program for this purpose ^29^. The best poses were manually inspected using Pymol and Discovery Studio ^30^.

### Statistical analysis

Quantitative data were submitted to the Shapiro-Wilk normality test. Parametric data were submitted to the ANOVA test, followed by Tukey, and expressed as Mean±SEM. Non-parametric data were submitted to the Kruskal-Wallis test, followed by Dunn, and expressed as a median (minimum-maximum). All analyses were performed using the statistical software GraphPad Prism 6.0®, considering a significance level of 95% (p<0.05).

**Box 2:**
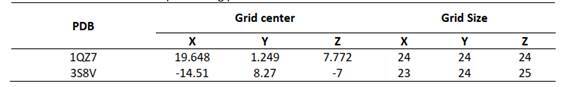
Box's dimensions and positioning parameters.

## Results

### Lithium Chloride mitigates bone loss potentiated by Monensin

**Figure 1:**
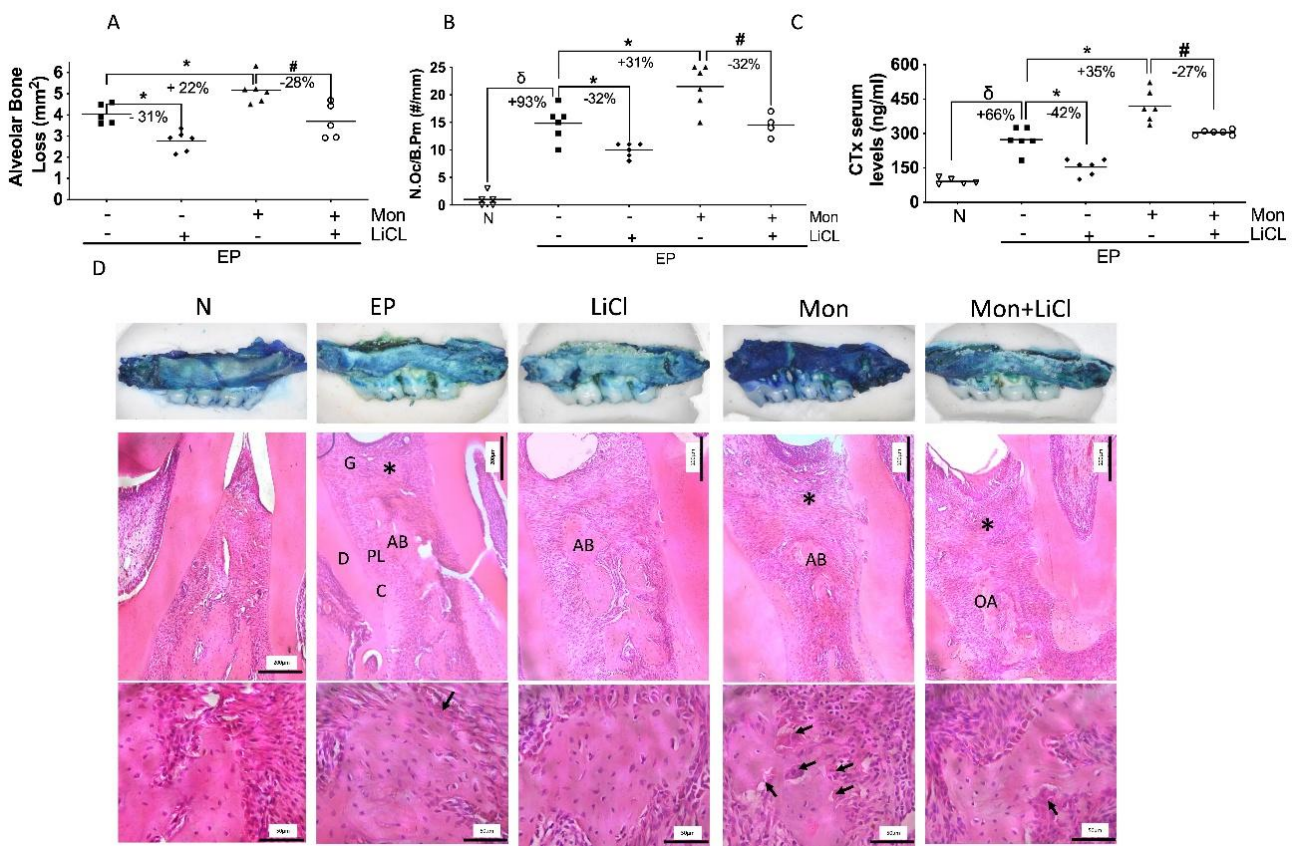
Lithium Chloride mitigates alveolar bone loss potentiated by Monensin**.** A) Alveolar bone loss; B) Osteoclast count/bone perimeter (N.Oc./B.Pm); C) Serum CTx levels; D) Macroscopic and Histological aspect of the periodontal tissue. (δ) indicates the statistical difference compared to the Naïve (N) group; (*) indicates the statistical difference compared to the Experimental Periodontitis (EP) group; (#) indicates the statistical difference compared to the Monensin (Mon) group. ANOVA and Tukey tests (p<0.05).

The model of experimental periodontitis (EP) induced by ligature was marked by bone loss, root exposure, and furcation lesion (Figures 1A and D), with an increase in osteoclast number (Figure 1B) and in CTx serum levels (Figure 1C). It was also seen as an important inflammatory infiltrate on the periodontium of these animals (Table 1). The treatment with LiCL significantly attenuated bone loss (Figures 1A and D) with a reduction in the number and function of osteoclasts (Figures 1B and C), compared to EP. LiCl also reduces periodontal inflammation (Box 1). Meanwhile, Monensin potentiated bone loss (Figure 1D), increasing the number of osteoclasts (Figure 1A) as well as CTx levels (Figure 1C) (p<0.05). A greater inflammatory infiltrate on periodontium was observed in these animals (Table 1). However, the use of LiCl in the group of animals with EP receiving Mon was able to significantly mitigate bone loss by 28% (p<0.05), reducing the number of osteoclasts by 32% (Figure 1B) and CTx serum levels by 27% (Figure 1C) compared to Mon group. LiCL also improved the periodontal tissue's histological aspects with Mon (Table 1). Taken together, LiCl was able to protect bone tissue by reducing osteoclast number and activity in animals with periodontitis receiving Monensin.

AB=alveolar bone; G=gingiva; D=dentin; PL=periodontal ligament; C=cementum; *=inflammatory infiltrate. Black arrows indicate Osteoclasts. 40X and 400x magnification, HE staining. Scale bar = 200µm and 50µm.

**Table 1:** Histopathological analysis of the periodontium.

Escore (0-3)	Experimental groups (n=6/group)				
	N	EP	Mon	LiCl	Mon+LiCl
(1) Absence of inflammatory infiltrate	0	0	0	0	0
(2) Discrete inflammatory infiltrate	0	0	0	4	1
(3) Moderate inflammatory infiltrate	0	2	1	2	4
(4) Intense inflammatory infiltrate	0	4	5	0	1
Median (extreme values)	0 (0-0)	3(2-3)^δ^	3(2-3)	1(1-2)*	2(1-3)^#^

Values are presented in an absolute number of animals/score and the median (extreme values).

N = Naïve; PE = experimental periodontitis; LiCl = Lithium chloride; Mon = Monensin.

(^δ^) Indicates the statistical difference compared to the Naïve group (N);

(*) Indicates the statistical difference compared to the experimental periodontitis (EP) group;

(^#^) Indicates statistical difference compared to Monensin (Mon).

Kruskal-Wallis test followed by Dunn (p<0,05).

### Lithium chloride rescues the deleterious effect of Monensin on osteoblasts.

EP significantly reduced the number of osteoblasts in the periodontal tissue (Figures 2A and D) (p<0.05). LiCL reversed the low number of osteoblasts (Figure 2A) with the increase in Runx2 and OPG gene expression compared to EP (p<0.05). Monensin significantly reduced the number and function of osteoblasts (Figure 2A-C). On the other hand, when LiCl was used in the animals with EP receiving Mon, it was seen an increase in osteoblast count by 39%, marked by an increase in Runx2 (+70%) and OPG (+68%) gene expressions. Confirming that LiCl was effective in stimulating osteoblasts even in animals with periodontitis receiving Mon.

### Lithium chloride counteracts Mon-induced Wnt signaling inhibition

Considering that Wnt signaling plays an important role in osteoblastogenesis and function and that LiCl and Mon are modulators of Wnt signaling, we have decided to investigate the behavior of the Wnt pathway under the administration of LiCl + Mon.

**Figure 3:**
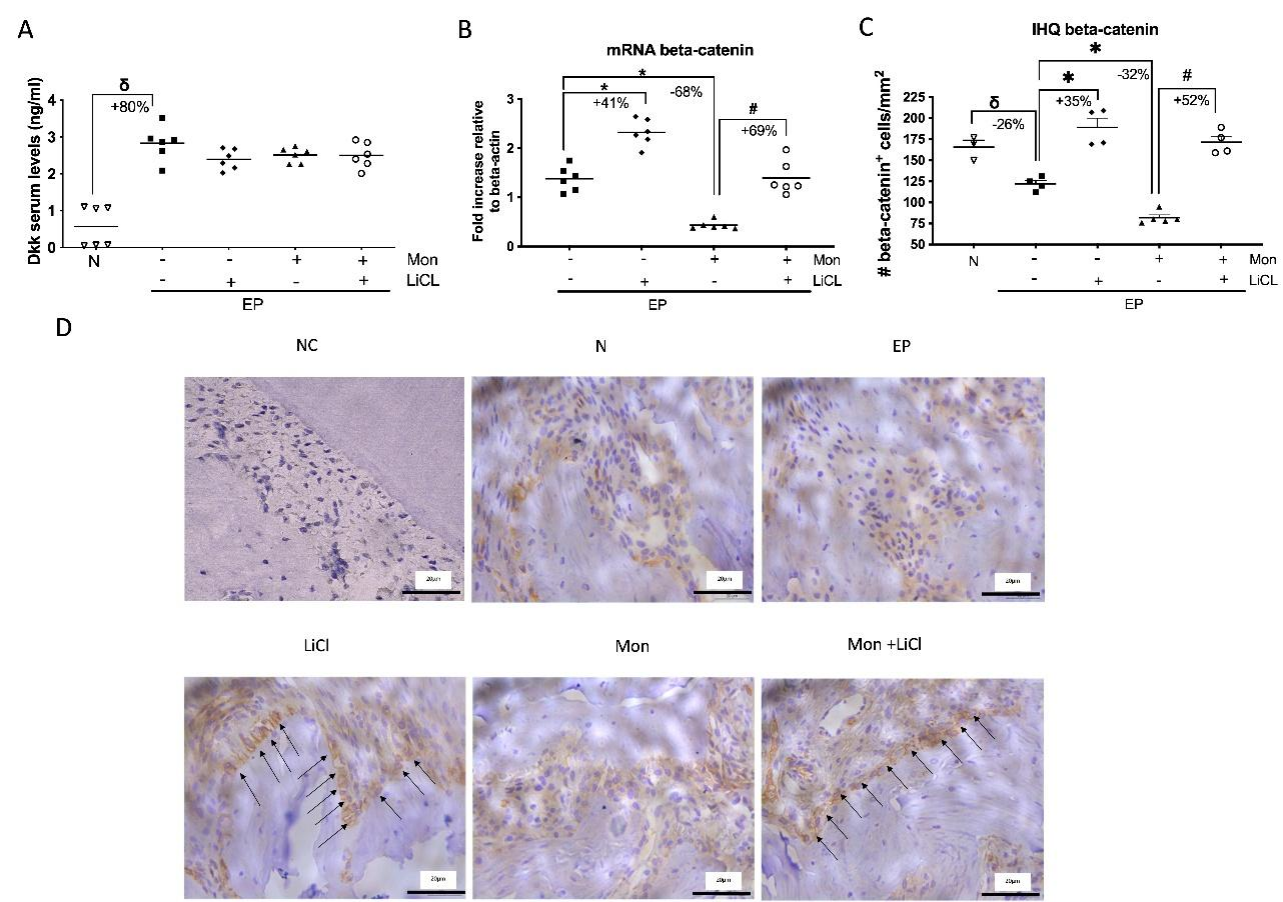
Lithium Chloride stimulates Wnt signaling reactivation. A) Serum DKK-1 levels; B) Beta-catenin mRNA expression; C) Immunopositive for beta-catenin in osteoblast cells count/mm^2^; D) Immunohistochemical aspect of hemimaxillae. ANOVA and Tukey tests. (p<0.05) (δ) indicates the statistical difference compared to the Naïve (N) group; (*) indicates the statistical difference compared to the experimental periodontitis (EP) group; (#) indicates the statistical difference compared to the Monensin (Mon) group. ANOVA and Tukey tests (p<0.05). Black arrows indicate immunopositive staining. 400x magnification. CN = Negative control. Scale bar = 20µm.

Dkk-1 is a Wnt antagonist, and our group has already shown its role in periodontal bone loss ^9^. However, there was no difference between the groups treated with either LiCl and/or Mon (p>0.05) (Figure 3A), indicating their lack of action on this component.

Beta-catenin is an effector key molecule of the Wnt pathway. LiCl increased the expression of both the Beta-catenin gene and protein (Figures 3B-D). Mon drastically reduced the expression of both genes and proteins. The use of LiCl in animals with EP receiving Mon restored the expression of Beta-catenin compared to the Mon group.

**igure 2:**
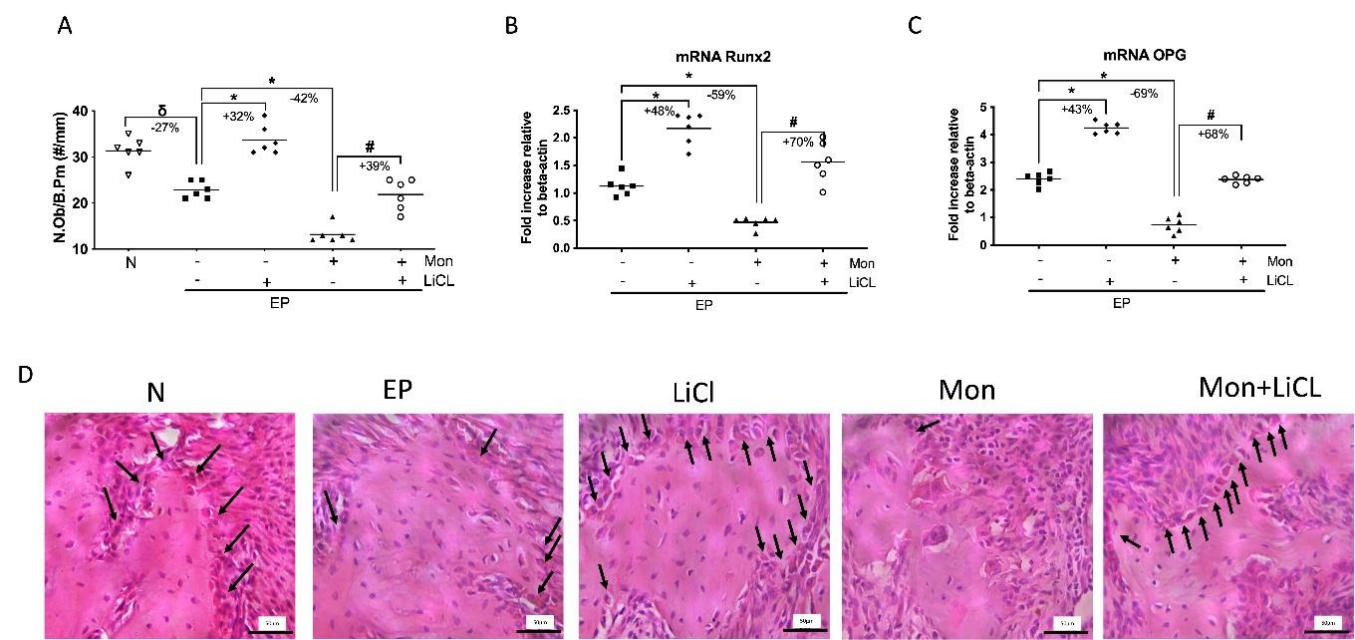
Lithium Chloride rescues the deleterious effect of periodontitis and Monensin on osteoblasts**.** A) Osteoblast count/bone perimeter (N.Ob./B.Pm); B) Runx2 mRNA expression; C) OPG mRNA expression ; D) Histological aspect of the periodontal tissue. (δ) indicates the statistical difference compared to the Naïve (N) group; (*) indicates the statistical difference compared to the Experimental Periodontitis (EP) group; (#) indicates the statistical difference compared to the Monensin (Mon) group. ANOVA and Tukey tests (p<0.05). Black arrows indicate Osteoblasts. 400x magnification, HE staining.Scale bar = 50µm.

### The molecular interaction mode between monensin and the β-catenin and LRP6 proteins

**Figure 4:**
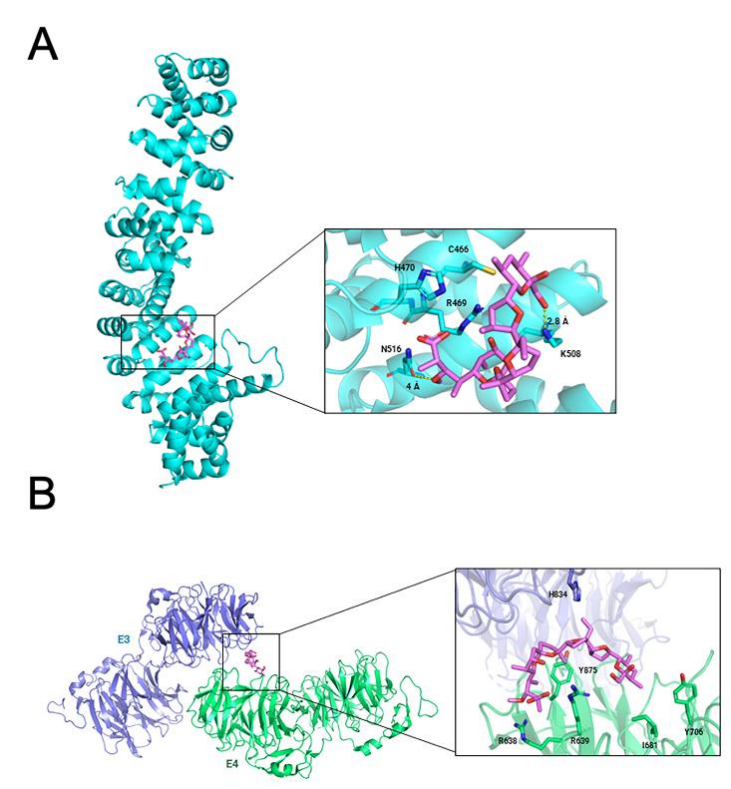
Molecular interaction of Monensin with Wnt pathway. A) Binding mode between β-catenin (cyan) and Monensin (pink); B) Binding mode LRP6 and Monensin

Initially, concerning the β-catenin protein, poses 12 and 9 assumed by Monensin were identified as the most favorable at the binding site, displaying binding energies of -9.3 kcal/mol and -7.5 kcal/mol, respectively. All poses exhibited the same conformation, with an RMSD of 0.3 Å. Therefore, the pose 12 was chosen (Figure 4A). Monensin binds to an exposed cavity located within the armadillo repeat domain. Within this site, crucial interactions occur with the residues Lys508 and Arg469. Lys508 forms a hydrogen bond with the oxygen adjacent to the carbon of the ligand's pyran ring end, while Arg469 interacts with the adjacent oxygen of the ligand's central pyran ring. Additionally, the residues Cys429 and Cys466 establish alkyl-type interactions with the carbons linked to the furan and pyran rings.

In the LRP6 protein, it was observed that poses 2 and 7 were the most favorable, displaying binding energies of -8.2 kcal/mol and -7.8 kcal/mol, respectively. These poses showed different conformations, with an RMSD of 2.7 Å. Following a manual analysis, pose 2 was selected (Figure 4B). Monensin binds to the interface of the E3 and E4 domains, establishing hydrogen interactions between the carbonyl oxygens and the adjacent oxygen, specifically with Arg638 and Arg639. Additionally, alkyl interactions occur between the pyran ring carbon at the end and the residues Ile681 and Tyr706, along with π-alkyl interactions involving residues His834, Tyr875, and Ala640.

## Discussion

In this study, the model of ligature-induced periodontitis was effective due to the intense bone resorption caused, confirming the previous findings of our group ^8^ ^,^ ^21^ ^,^ ^22^ ^,^ ^31^ ^);^ ^32^ ^;^ ^33^. LiCl, a Wnt agonist, protected bone tissue, reduced osteoclast count, and increased the number of osteoblasts. Mon, a Wnt antagonist, has potentiated bone loss and inflammation, which is marked by the increase in osteoclasts and reduction in osteoblast counts, also affecting their function; however, when the use of LiCl in animals with periodontitis receiving Monensin was able to reverse their deleterious effect on bone tissue.

In this study, Mon potentiated bone loss induced by periodontitis. Mon is an ionophore antibiotic recently indicated as a drug with anti-cancer action ^12^. This is precisely because it inhibits the canonical WNT pathway by blocking the LRP5/6 receptor and beta-catenin ^16^. Molecular docking analyses revealed the high affinity of Monensin for the above-mentioned proteins. Furthermore, it was identified that Monensin forms a hydrogen bond with Lys508 of beta-catenin, a hotspot known to interact with other inhibitors ^34^. With the LRP6 receptor, it was observed that Monensin interacts with residues Ile681, Tyr706, and Tyr875, components of a hydrophobic patch crucial for interactions with its biological inhibitor, DKK-1 ^35^ ^,^ ^36^.

In bone tissue, the blockage of Wnt is related to a reduction in OPG, leading to a higher interaction between RANK/RANKL ^37^, improving osteoclastogenesis, and bone resorption ^8^, as confirmed by our findings. However, to the best of our knowledge, this is the first time that the effect of Monensin has been evaluated on periodontal bone loss.

As demonstrated, LiCl protected the bone tissue of animals submitted to periodontitis. This agent has been used to treat mental disorders, but considering its inhibition of GSK3b ^38^ ^,^ ^39^ ^,^ ^40^ ^,^ ^41^, LiCl induces WNT pathway activation. In bone tissue, the activation of WNT signaling causes GSK3b inhibition, allowing the accumulation of beta-catenin in the cytoplasm, which then gains access to the nucleus and stimulates the expression of genes such as Runx2, the main transcription factor of osteoblasts and OPG, a marker of osteoblast function ^42^ corroborating our findings.

Previous studies in the periodontium have confirmed our findings regarding the osteoprotective effect of LiCl. It has been described that LiCl reduces ligature-induced bone loss in estrogen-deficient rats, improving the trabecular area with high bone marker expression ^18^. In an orthodontic tooth movement model, LiCl reduced root resorption and minimized periodontal ligament cell death ^43^. Lithium accelerated healing of apical periodontitis in an animal model ^40^ ^,^ ^44^ showed that LiCl caused an increase in osteogenic markers, such as Runx2 and Osterix.

Acknowledging the great effects of LiCl and Mon on bone tissue, we have decided to investigate if LiCl could rescue the monensin-potentiated bone loss in animals with periodontitis. This study showed that LiCl reversed bone loss, reducing osteoclast counts and CTx serum levels. Meanwhile, it restored the number and activity of osteoblasts in animals with periodontal bone loss potentiated by Monensin. It is noteworthy that other types of GSK3b inhibitors, such as BIO and CHIR99021, were unable to reverse the inhibition of Wnt signaling caused by Mon ^16^

To explain these findings and consider that LiCL and Mon directly interact with Wnt signaling, molecular assays were performed. Dkk-1 is an antagonist of the Wnt pathway, stimulated by inflammation, and has been reported to contribute to inflammatory bone ^8^. Despite the high levels of Dkk-1 in animals with periodontitis, there was no change after the treatments, indicating that neither LiCl nor Mon has Dkk-1 as a target.

**Figure 5:**
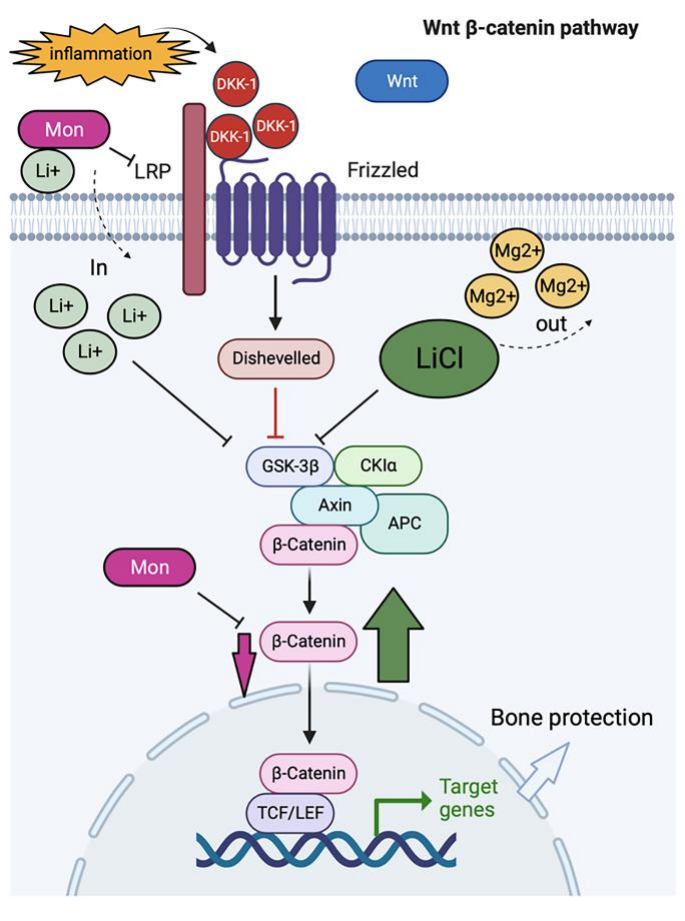
Proposed mechanism for LiCl to rescue Wnt signaling in animals receiving Monensin under an inflammatory condition.

Downstream, the pathway to the role of beta-catenin was evaluated, and both genetic and protein expressions were restored by using LiCl in animals with periodontitis receiving Mon ^16^ ^,^ ^45^. It has been reported that Mon inhibits beta-catenin, but LiCl can provide a greater beta-catenin expression. LiCl directly competes with magnesium ions for the binding site of this GSK3b, promoting its inhibition ^46^ and also can, indirectly, through phosphorylation in the Ser9 amino acid, act as a pseudosubstrate, inactivating GSK3b ^47^ Taken together, both direct and indirect effect of LiCl inhibiting GSK3b may stimulate a greater beta-catenin accumulation rescuing Wnt pathway and protecting bone tissue. Moreover, as an ionophore, Monensin can bind to Na+, K+, and Li+, facilitating their entry into the cell ^48^ ^,^ ^49^ ^,^ ^50^. Thus, we suggest that Mon favors the entry of Li+ into the cell, potentiating GSK3b inhibition with beta-catenin accumulation (Figure 5). However, more studies are needed to confirm this hypothesis.

In summary, this study's results showed that LiCl significantly mitigated bone loss potentiated by Monensin in experimental periodontitis due to a strong inhibition of GSK3b. Therefore, LiCl can be an important pharmacological tool to restore Wnt activation when this pathway has been intensively blocked.
